# Quantifying Intubation Forces on Incisors and Tongue Base Across Exposure Difficulty and Experience in a Simulator

**DOI:** 10.7759/cureus.41611

**Published:** 2023-07-09

**Authors:** Gavin Davis, Ronit E Malka, Austin Moore, Stacy L Cook, Megan Blackburn, Gregory R Dion

**Affiliations:** 1 Ophthalmology, Brooke Army Medical Center, San Antonio, USA; 2 Otolaryngology - Head and Neck Surgery, Brooke Army Medical Center, San Antonio, USA; 3 Hemorrhage and Edema Control, United States Army Institute of Surgical Research, San Antonio, USA; 4 Otolaryngology, University of Cincinnati Medical Center, Cincinnati, USA

**Keywords:** difficult laryngoscopy, direct laryngoscopy, endotracheal intubation, medical education, airway management

## Abstract

Objective: Laryngoscopy simulators quantifying forces on critical structures in progressively challenging airways and operator expertise are lacking. We aimed to quantify laryngoscopy forces across expertise and exposure difficulty.

Study design: Prospective observational study

Setting: Tertiary care medical center

Methods: Force gauges were affixed to a difficult airway mannequin to quantify teeth and tongue forces across increasingly challenging airway exposure. Medical students (*n*=10), residents (*n*=11), and otolaryngology staff (*n*=10) performed direct laryngoscopy using a Miller size 3 laryngoscope with 1) normal neck/jaw mobility, 2) restricted neck extension, 3) restricted jaw opening, and 4) restricted neck/jaw mobility. Incisor and tongue pounds of force (lbf) were continuously measured.

Results: As the difficulty setting increased, forces exerted by the students, residents, and staff on the incisors and tongue base increased (p=0.01). Between normal and maximally restricted settings, force delivered to the incisors increased by 6.95 lbf (standard error (SE) 1.29), 5.93 lbf (SE 0.98), and 5.94 lbf (SE 0.70) for the students, residents, and staff, respectively. At the tongue base, force increased by 0.37 lbf (SE 0.18), 0.46 lbf (SE 0.14), and 0.73 lbf (SE 0.15) for the students, residents, and staff, respectively. Esophageal intubations occurred in 50% of the students, 23% of the residents, and 45% of the otolaryngology staff at maximal difficulty, with none at the easiest setting (p=0.33). Compared to the residents, the staff applied significantly increased pressure on the tongue base during laryngoscopy (p=0.02).

Conclusion: Forces exerted on the incisors and tongue base varied across exposure difficulty and expertise levels, suggesting that they may be useful markers for training and competence assessment.

## Introduction

Direct laryngoscopy, either in the setting of otolaryngologic procedures or during intubation, is one of the most commonly performed procedures in the nation. In the United States alone, more than 15 million operating room intubations and one million hospital intubations occur each year [1]. Depending on the setting, individuals ranging from experienced attending physicians to novice emergency medical technicians (EMTs) may be tasked with this critical responsibility. This was highlighted during the COVID-19 pandemic, overwhelming hospitals with patients requiring emergent airway management and mechanical ventilation and further increasing the prevalence and relevance of this intervention [2].

Two of the most common complications associated with direct laryngoscopy are dental trauma and tongue paresthesia, both due to excess force applied during laryngoscopy. The incidence of dental injuries during endotracheal intubations has been shown to be as common as one in 150 intubations, with a majority involving the maxillary central incisors (51.8%) [3,4]. According to the literature, during direct operative laryngoscopy performed by otolaryngologists, the rate of dental injuries has ranged from 0.4% to 6.5% [5-8]. Tongue paresthesia and accompanying dysgeusia are also relatively common, with up to 12-15% of patients reporting tongue numbness and anywhere between 3% and 18% reporting dysgeusia postoperatively [9,10].

Direct laryngoscopy has traditionally been a challenging skill to learn and maintain, requiring more than 75 attempts to reach proficiency [11,12]. In particular, for novice users, direct laryngoscopy has been associated with a higher rate of oropharyngeal and dental trauma, with incidence of adverse events shown to increase proportionally with the number of attempts to secure an airway [13]. In addition, successful endotracheal intubation depends largely on experience, with EMTs, paramedics, and physicians having first-attempt failure rates up to 50%, 10-20%, and 10-15%, respectively [14-18]. Recent evidence suggests that low-fidelity simulators excel at task familiarization and early skill development, occupying a vital role in the early learning of technical medical tasks [19]. For this reason, mannequins have been used in airway management education for over 50 years [20-25]. While these traditional models exist to allow medical learners practice in direct laryngoscopy, they do not quantify forces applied and may reinforce poor habits. Recent work has focused on developing models that include measuring and assessing force application during direct laryngoscopy, both for intubation and operative laryngoscopy applications [26-31]. However, despite these models’ development, data describing force application during direct laryngoscopy as learners progress from novice to expert are lacking. This study aims to employ a replicable, easily manufactured model for direct laryngoscopy and quantify differences in intubation-related forces exerted on the teeth and base of the tongue across experience levels.

## Materials and methods

Model design and manufacture

**Figure 1:**
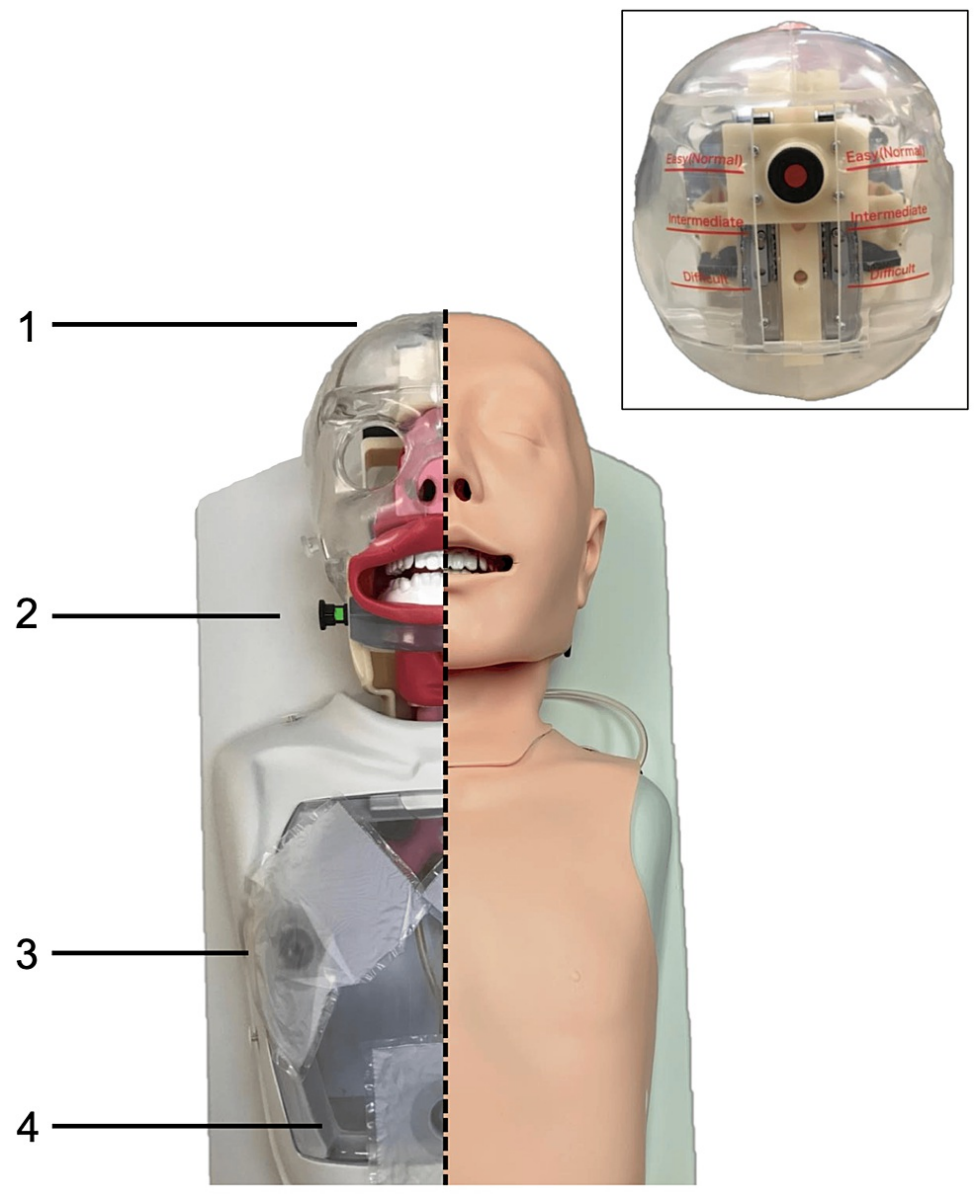
Commercially available mannequin for the direct laryngoscopy and intubation practice as seen during use on the right and with silicone cover removed on the left, demonstrating controls for adjusting (1) the neck extension and (2) jaw opening, (3) lung inflation to confirm successful intubation, and (4) stomach inflation to demonstrate unsuccessful esophageal intubation. Inset shows neck extension control.

**Figure 2:**
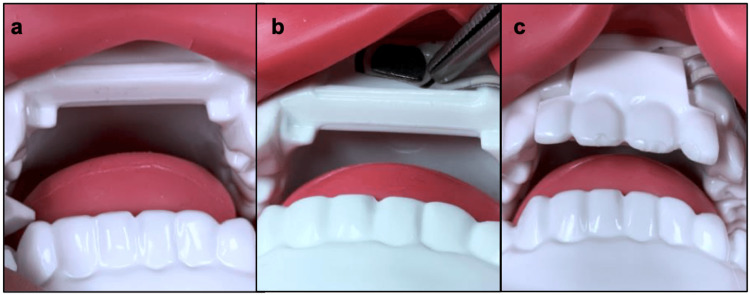
Force gauge placement on the mannequin, demonstrating (a) removal of mannequin incisors, (b) placement of the force gauge on the incisor, (c) replacement of incisors over the force sensor, and (d) placement of the force sensor along the laryngoscope.

A commercially available airway simulation mannequin (Difficult Airway Management Simulator, Kyoto Kagaku, Kyoto, Japan) was modified for applied force measurements at the tongue base and teeth during direct laryngoscopy. The mannequin has prefabricated settings to independently increase neck rigidity across two settings (normal and rigid) and jaw rigidity across three settings (normal, impaired, and trismic; see Figure 1). To measure force at the incisors, force gauges (FlexiForce B201 0-25lb, Tekscan, Boston, Massachusetts, USA) were inset into the base of the mannequin’s upper incisors (Figure 2). To measure tongue base forces, identical force gauges were placed 4 cm distal to the tip of a Miller size 3 laryngoscope, correlating to the tongue base when the scope was appropriately placed to achieve adequate visualization of the vocal folds. Both force gauges were calibrated within 24 hours of use with the Mark-10 Force Gauge (Mark-10, Copiague, New York, USA). The force measurements generated during direct laryngoscopy were recorded on a laptop computer using the Economic Load Force Measurement System (ELF) software from Tekscan (Massachusetts, USA).

Participant recruitment

This project was reviewed by the Institutional Review Board (IRB) of the 59th Medical Wing Institutional Review Board, United States Army. The participants were recruited from the otolaryngology department of a tertiary academic medical center. These participants were unpaid volunteers and informed that they would be intubating a mannequin at various difficulty settings for a study dedicated to the validation of a training mannequin.

Direct laryngoscopy force measurements

This study examined three groups of participants with varying levels of airway management experience. The three groups were defined as medical students (less than six months of laryngoscopy experience), residents (six months-five years of experience), and otolaryngology staff (greater than five years of experience). Before starting, each participant was educated on the four different difficulty settings and informed of the pressure sensors on the laryngoscope and under the incisors. The participants were instructed that their goal was to successfully intubate the mannequin without time limit, with success confirmed by inflation of the mannequin lungs through the placed endotracheal tube after intubation. The procedure bed was adjusted to ensure proper ergonomics for the participant, and the mannequin was lubricated with TruCorp® lubricant between each user to ensure ease of use and protect the mannequin materials. For standardization, all subjects were allowed one practice intubation at the normal setting to familiarize themselves with the mannequin before starting the eight measured attempts. All subjects were also required to use a Miller 3 blade and place a 7.0 mm cuffed endotracheal tube (Covidien, Dublin, Ireland).

The Difficult Airway Management Simulator (training model) was manufactured to have 24 possible variations of difficulty, including one normal setting, three stages of mouth opening, two stages of neck ﬂexibility, two tongue sizes, and two positions of the vocal cords. In this study, only the jaw and neck extension variables were manipulated. Each participant attempted to intubate a mannequin two times, each in four different airway configurations sequentially: 1) normal neck and jaw mobility, 2) restricted neck extension, 3) restricted jaw opening, and 4) restricted neck and jaw mobility.

For each participant, the primary outcomes of force delivered to the upper incisors and laryngoscope during intubation were measured at a sampling frequency of 200 Hz throughout the intubation attempts. The secondary outcomes of the intubation success and dislodgement of the teeth were additionally recorded.

Statistical analysis

The maximum and average force measurements were compared using Wilcoxon rank sum tests for unpaired numerical data and multiple-comparison contingency chi-square test for categorical data. Linear regression was performed for all intragroup comparisons. All statistical analyses were conducted within MATLAB version R2021A with the Statistical Toolbox (Mathworks Inc., Natick, Massachusetts, USA).

## Results

Force on incisors

Regardless of experience, all the participant groups had the lowest maximal and average applied force to the incisors with normal jaw and neck mobility and highest applied force with restriction in both jaw and neck mobility (Tables 1-2). The average maximal force applied to the incisors at normal settings was 3.64 lbf (standard error (SE) 0.71) for the medical students, 2.21 lbf (SE 0.40) for the residents, and 1.41 lbf (SE 0.27) for the attending physicians. With restricted jaw and neck movement, these rose to 10.60 lbf (SE 1.51) for the medical students, 7.70 lbf (SE 1.11) for the residents, and 7.43 lbf (SE 0.65) for the attending physicians and increased over normal mobility forces for all cohorts (p<0.001 for all comparisons). Similarly, the average force increased from 1.01 lbf (SE 0.22) for the medical students, 0.77 lbf (SE 0.18) for the residents, and 0.49 lbf (SE 0.08) for the attending physicians at normal settings to 4.24 lbf (SE 0.64), 3.26 lbf (SE 0.63), and 3.10 lbf (SE 0.32), respectively, with maximally restrictive settings (p<0.001 for all comparisons). The attending physicians also demonstrated significantly increased maximal (1.37 lbf (SE 0.28)) and average (0.70 lbf (SE 0.15)) force on the incisors with decreased jaw opening or neck extension (p=0.01). There was no difference in the maximal or average force exerted between the restricted neck and jaw opening settings across all groups. All intragroup comparisons across difficulty settings are summarized in Table 3 for the maximum force data and Table 4 for the average force data.

**Table 1:** Maximum force applied to the incisors and laryngoscope corresponding to the location of the tongue base during all laryngoscopy trials, stratified by mannequin setting and user experience. Average maximum force (Fm) and standard error (SE) reported. All measurements denoted in pounds of force (lbf).

	Students (*n*=10)		Residents (*n*=11)		Attending physicians (*n*=10)	
	Incisors F_m_ (SE)	Scope F_m_ (SE)	Incisors F_m_ (SE)	Scope F_m_ (SE)	Incisors F_m_ (SE)	Scope F_m_ (SE)
Normal neck and jaw mobility	3.64 (0.71)	1.59 (0.23)	2.21 (0.40)	0.91 (0.10)	1.41 (0.27)	1.40 (0.13)
Restricted neck extension	4.38 (0.74)	1.45 (0.27)	3.27 (0.61)	1.10 (0.17)	2.73 (0.34)	2.21 (0.33)
Restricted jaw opening	5.50 (1.07)	2.42 (0.39)	3.24 (0.76)	1.57 (0.37)	2.89 (0.61)	2.23 (0.29)
Restricted neck and jaw mobility	10.60 (1.51)	3.03 (0.51)	7.70 (1.11)	2.34 (0.41)	7.43 (0.65)	4.85 (0.72)

**Table 2:** Average force applied to the incisors and laryngoscope corresponding to the location of the tongue base during all laryngoscopy trials, stratified by mannequin setting and user experience. Average force (Favg) and standard error (SE) reported. All measurements denoted in pounds of force (lbf).

	Students (*n*=10)		Residents (*n*=11)		Attending physicians (*n*=10)	
	Incisors F_avg_ (SE)	Scope F_avg_ (SE)	Incisors F_avg_ (SE)	Scope F_avg_ (SE)	Incisors F_avg_ (SE)	Scope F_avg_ (SE)
Normal neck and jaw mobility	1.01 (0.22)	0.59 (0.09)	0.77 (0.18)	0.36 (0.04)	0.49 (0.08)	0.52 (0.05)
Restricted neck extension	1.53 (0.21)	0.47 (0.07)	1.75 (0.39)	0.37 (0.05)	1.18 (0.16)	0.77 (0.11)
Restricted jaw opening	1.73 (0.44)	0.80 (0.15)	1.20 (0.28)	0.54 (0.09)	0.78 (0.14)	0.73 (0.09)
Restricted neck and jaw mobility	4.24 (0.64)	0.98 (0.18)	3.26 (0.63)	0.85 (0.18)	3.10 (0.32)	1.27 (0.13)

**Table 3:** Intragroup comparison of the maximum force applied to the incisors and laryngoscope during all trials, stratified by difficulty setting and user experience. Mean and median difference in maximum force in pounds of force (lbf) with associated p-values noted, with standard error (SE) and range in parentheses.

	Incisors			Laryngoscope		
Group	Mean difference (SE)	Median difference (range)	p	Mean difference (SE)	Median difference (range)	p
Student						
Normal mobility vs. restricted neck extension	0.73 (0.60)	0.50 (-3.25, 7.08)	0.25	--0.15 (0.34)	0.00 (-3.21, 3.64)	0.41
Normal mobility vs. restricted jaw opening	1.85 (0.75)	1.38 (-5.77, 10.47)	0.12	0.83 (0.43)	0.34 (-3.04, 4.95)	0.10
Normal mobility vs. restricted neck/jaw mobility	6.95 (1.29)	6.72 (-2.65, 21.77)	<0.001	1.38 (0.50)	1.01 (-1.76, 7.80)	0.01
Restricted neck extension only vs. restricted jaw opening only	1.11 (0.72)	0.59 (-3.5,7.74)	0.75	0.97 (0.42)	0.39 (-2.21, 4.95)	0.04
Resident						
Normal mobility vs. restricted neck extension	1.09 (0.57)	1.00 (-3.37, 8.82)	0.22	0.19 (0.14)	0.00 (-0.390, 2.96)	0.67
Normal mobility vs. restricted jaw opening	1.00 (0.85)	0.69 (-5.57, 11.61)	0.48	0.66 (0.31)	0.00 (-0.20, 5.06)	0.28
Normal mobility vs. restricted neck/jaw mobility	5.93 (0.98)	5.04 (-1.21, 13.78)	<0.001	1.36 (0.37)	1.04 (-0.29, 5.20)	0.005
Restricted neck extension only vs. restricted jaw opening only	0.09 (0.62)	0.24 (-7.89, 3.56)	0.58	0.42 (0.28)	0.00 (-0.78, 4.81)	0.50
Attending						
Normal mobility vs. restricted neck extension	1.37 (0.28)	1.35 (-1.04, 3.16)	0.01	0.80 (0.34)	0.48 (-0.84, 5.07)	0.06
Normal mobility vs. restricted jaw opening	1.31 (0.49)	0.78 (-1.03, 7.60)	0.08	0.82 (0.34)	0.48 (-0.80, 6.11)	0.008
Normal mobility vs. restricted neck/jaw mobility	5.94 (0.70)	6.35 (1.43, 11.66)	<0.001	3.42 (0.78)	2.09 (0.65, 13.33)	<0.001
Restricted neck extension only vs. restricted jaw opening only	0.16 (0.51)	0.24 (-3.90, 6.24)	0.85	0.02 (0.29)	0.11 (-4.16, 2.29)	0.73

**Table 4:** Intragroup comparison of the average force applied to the incisors and laryngoscope during all trials, stratified by difficulty setting and user experience. Mean and median difference in average force in pounds of force (lbf) with associated p-values noted, with standard error (SE) and range in parentheses.

	Incisors			Laryngoscope		
Group	Mean difference (SE)	Median difference (range)	p	Mean difference (SE)	Median difference (range)	p
Student						
Normal mobility vs. restricted neck extension	0.52 (0.26)	0.49 (-1.93, 2.93)	0.01	-0.13 (0.11)	0.02 (-0.97, 1.31)	0.64
Normal mobility vs. restricted jaw opening	0.72 (0.41)	0.27 (-1.79, 6.47)	0.17	0.21 (0.15)	0.07 (-1.01, 1.44)	0.31
Normal mobility vs. restricted neck/jaw mobility	3.23 (0.55)	2.66 (0.25, 8.78)	<0.001	0.37 (0.18)	0.18 (-1.13, 2.24)	0.15
Restricted neck extension only vs. restricted jaw opening only	0.20 (0.39)	-0.21 (-2.38, 6.20)	0.23	0.33 (0.14)	0.05 (-0.37, 1.94)	0.10
Resident						
Normal mobility vs. restricted neck extension	1.02 (0.31)	0.52 (-0.72, 5.32)	0.01	0.01 (0.04)	-0.01 (-0.42, 0.76)	0.82
Normal mobility vs. restricted jaw opening	0.43 (0.29)	0.27 (-2.44, 3.70)	0.04	0.18 (0.06)	0.04 (-0.05, 0.70)	0.15
Normal mobility vs. restricted neck/jaw mobility	2.66 (0.56)	2.00 (0.01, 9.67)	<0.001	0.46 (0.14)	0.15 (-0.30, 2.05)	0.01
Restricted neck extension only vs. restricted jaw opening only	-0.57 (0.42)	-0.05 (5.53, 1.91)	0.28	0.16 (0.08)	0.09 (-0.52, 1.11)	0.21
Attending						
Normal mobility vs. restricted neck extension	0.70 (0.15)	0.52 (-0.22, 1.89)	0.002	0.25 (0.11)	0.13 (-0.50, 1.73)	0.14
Normal mobility vs. restricted jaw opening	0.25 (0.10)	0.13 (-0.28, 1.68)	0.15	0.19 (0.08)	0.05 (-0.32, 1.04)	0.12
Normal mobility vs. restricted neck/jaw mobility	2.60 (0.36)	2.28 (0.42, 5.73)	<0.001	0.73 (0.15)	0.61 (-0.23, 2.89)	<0.001
Restricted neck extension only vs. restricted jaw opening only	-0.39 (0.13)	-0.34 (-1.32, 0.70)	0.07	-0.05 (0.08)	0.01 (-0.93, 0.50)	0.97

At normal settings, the attending physicians applied significantly less pressure on the incisors than the medical students (2.38 lbf (SE 0.83) less by the attending physicians; p=0.02). While more experienced laryngoscopists applied lower maximal and average force to the incisors across the settings, these differences did not reach statistical significance. All intergroup comparisons across the difficulty settings are summarized in Table 5 for the maximum force data and Table 6 for the average force data.

**Table 5:** Intergroup comparison of the maximum force applied to the incisors and laryngoscope during all trials, stratified by difficulty setting and user experience. Difference in mean maximum force in pounds of force (lbf) with associated p-values noted, with standard error (SE) and range denoted in parentheses. Negative value denotes less force exerted by the second group compared to the first.

	Incisors			Laryngoscope		
Group	Mean difference (SE)	Median difference (range)	p	Mean difference (SE)	Median difference (range)	p
Normal neck and jaw mobility						
Student vs. resident	-1.35 (1.00)	-0.31 (-9.95, 3.85)	0.20	-0.67 (0.26)	-0.59 (-2.87, 0.89)	0.01
Student vs. attending	-2.38 (0.83)	-1.98 (-10.06, 2.95)	0.02	-0.25 (0.28)	0.06 (-3.25, 1.78)	0.94
Resident vs. attending	-0.66 (0.47)	-0.63 (-4.97, 2.52)	0.20	0.50 (0.16)	0.41 (-0.40, 1.94)	0.005
Restricted neck extension						
Student vs. resident	0.65 (1.01)	-0.91 (-12.26, 6.76)	0.15	-0.50 (0.30)	-0.22 (-4.03, 1.07)	0.23
Student vs. attending	-1.65 (0.89)	-0.66 (-13.84, 2.82)	0.18	0.76 (0.42)	0.83 (-3.98, 4.17)	0.01
Resident vs. attending	-0.63 (0.84)	0.51 (-8.79, 4.25)	0.89	1.26 (0.41)	0.94 (-1.77, 5.83)	0.001
Restricted jaw opening						
Student vs. resident	-1.82 (1.44)	-1.14 (-11.96, 9.65)	0.06	-1.13 (0.46)	-0.46 (-4.61, 1.96)	0.03
Student vs. attending	-2.60 (1.30)	-0.66 (-16.99, 7.24)	0.09	-0.19 (0.36)	0.21 (-3.17, 2.56)	0.76
Resident vs. attending	-0.32 (1.05)	-0.11 (-12.35, 4.54)	0.69	0.86 (0.53)	1.00 (-4.31, 6.73)	0.01
Restricted neck and jaw mobility						
Student vs. resident	-2.94 (2.14)	-1.60 (-22.90, 8.10)	0.12	-1.03 (0.67)	-0.78 (-7.93, 4.27)	0.23
Student vs. attending	-3.58 (1.53)	-3.57 (-21.38, 4.09)	0.15	1.57 (0.97)	1.56 (-7.54, 12.44)	0.02
Resident vs. attending	0.05 (1.67)	2.86 (-12.82, 9.68)	0.64	2.48 (0.90)	1.92 (-3.41, 11.65)	0.006

**Table 6:** Intergroup comparison of the average force applied to the incisors and laryngoscope during all trials, stratified by difficulty setting and user experience. Difference in mean maximum force in pounds of force (lbf) with associated p-values noted, with standard error (SE) and range denoted in parentheses. Negative value denotes less force exerted by the second group compared to the first.

	Incisors				Laryngoscope		
Group	Mean difference (SE)		Median difference (range)	p	Mean difference (SE)	Median difference (range)	p
Normal neck and jaw mobility							
Student vs. resident		-0.21 (0.31)	-0.08 (-3.27, 1.78)	0.25	-0.22 (0.10)	-0.15 (-1.03, 0.53)	0.03
Student vs. attending		-0.55 (0.21)	-0.36 (-2.70, 0.66)	0.07	-0.09 (0.11)	-0.02 (-1.06, 0.64)	0.96
Resident vs. attending		0.32 (0.21)	-0.05 (-2.47, 1.20)	0.55	0.16 (0.07)	0.10 (-0.38, 0.70)	0.011
Restricted neck extension							
Student vs. resident		0.32 (0.51)	-0.13 (-3.38, 5.50)	0.37	-0.11 (0.10)	-0.07 (-1.46, 0.71)	0.08
Student vs. attending		-0.36 (0.26)	-0.29 (-2.84, 1.36)	0.26	0.31 (0.13)	0.19 (-1.03, 1.64)	0.02
Resident vs. attending		-0.67 (0.52)	0.11 (-5.28, 1.70)	0.74	0.42 (0.14)	0.29 (-0.80, 1.95)	0.001
Restricted jaw opening							
Student vs. resident		-0.24 (0.52)	0.09 (-5.36, 4.62)	0.54	-0.33 (0.22)	-0.15 (-2.06, 1.13)	0.13
Student vs. attending		-0.95 (0.50)	-0.21 (-7.58, 1.97)	0.09	-0.07 (0.13)	-0.02 (-1.09, 0.95)	0.66
Resident vs. attending		-0.50 (0.33)	-0.25 (-4.74, 1.00)	0.23	0.25 (0.14)	0.21 (-0.68, 1.27)	0.04
Restricted neck and jaw mobility							
Student vs. resident		-0.97 (1.05)	-1.81 (-9.55, 8.85)	0.13	-0.19 (0.29)	-0.16 (-2.78, 2.55)	0.48
Student vs. attending		-1.33 (0.64)	-0.54 (-8.93, 2.15)	0.23	0.24 (0.27)	0.26 (-2.30, 2.59)	0.07
Resident vs. attending		-0.15 (0.84)	0.62 (-9.19, 5.06)	0.36	0.43 (0.24)	0.78 (-1.50, 2.24)	0.02

Maximum force on the laryngoscope

**Figure 3:**
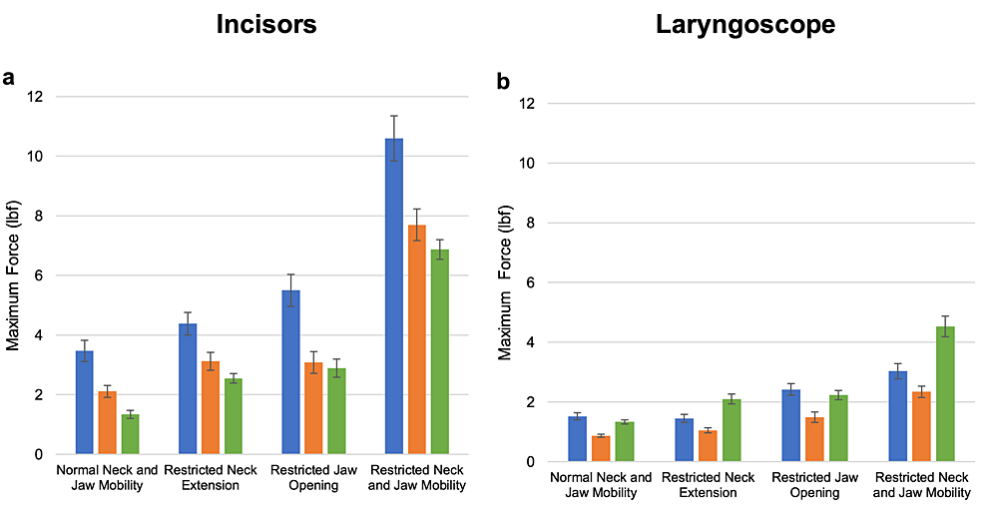
Maximum force applied to the incisors (a) and laryngoscope (b) averaged across all laryngoscopy trials, stratified by difficulty setting and user experience. Student data are shown in blue, resident data in orange, and attending physician data in green. Error bars denote the standard error.

**Figure 4:**
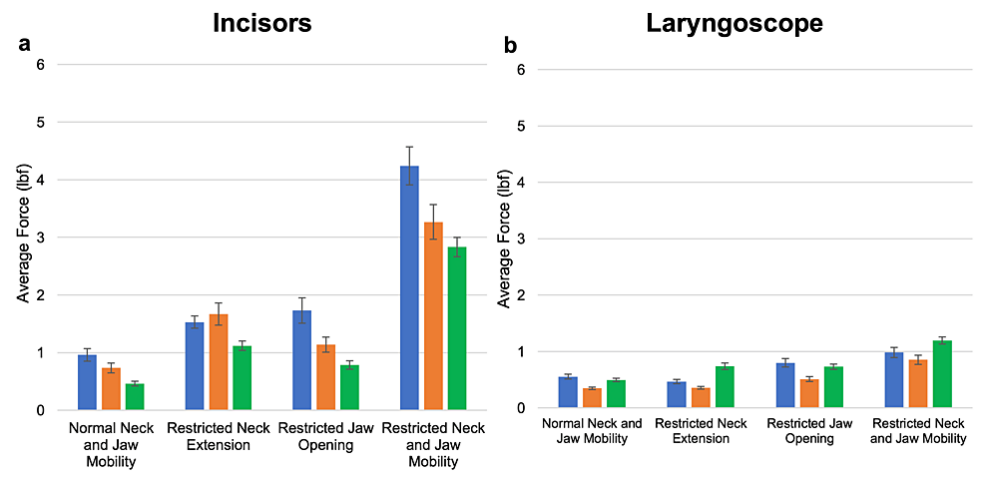
Average force applied to the incisors (a) and laryngoscope (b) averaged across all laryngoscopy trials, stratified by difficulty setting and user experience. Student data are shown in blue, resident data in orange, and attending physician data in green. Error bars denote the standard error.

Across all the groups, force applied to the distal aspect of the laryngoscope was lowest with normal jaw and neck mobility and highest applied force with restriction in both jaw and neck mobility (Tables 1-2). The maximal force applied to the distal end of the laryngoscope was significantly lower than the incisors for all comparisons, with an average of 1.59 lbf (SE 0.23) for the medical students, 0.91 lbf (SE 0.10) for the residents, and 1.1.41 lbf (SE 0.27) for the attending physicians at normal settings, rising to 3.03 lbf (SE 0.51), 2.34 lbf (SE 0.41), and 4.85 lbf (SE 0.72), respectively, at maximally restricted settings (p=0.01 or less for all comparisons). The average force also rose for all groups, from 0.59 lbf (SE 0.09) for the medical students, 0.36 lbf (SE 0.04) for the residents, and 0.52 lbf (SE 0.05) for the attending physicians at normal settings to 0.98 lbf (SE 0.18), 0.85 lbf (SE 0.18), and 1.27 lbf (SE 0.13), respectively, although this did not reach statistical significance for the medical students (p=0.01 or less for the residents and attending physicians). There was no difference in the maximal or average force exerted between the restricted neck and restricted jaw opening settings on the laryngoscope in any group (Tables 3-4). The maximum and average force applied to the incisors and laryngoscope averaged across all laryngoscopy trials, stratified by difficulty setting and user experience, are illustrated in Figure 3 and Figure 4.

The attending physicians applied more pressure on the tongue from the laryngoscope than the residents across all settings, ranging from 0.50 lbf (SE 0.16) at normal settings to 2.48 lbf (SE 0.90) at maximally restrictive settings (p=0.01 or less for all comparisons). In addition, the attending physicians applied more pressure than the medical students with restricted jaw opening and neck extension (1.57 lbf (SE 0.97); p=0.05). The residents applied on average less force than the medical students on the distal laryngoscope across all difficulty settings, although this reached statistical significance at the normal and reduced jaw opening settings (p=0.01).

Failed intubations

Failed intubation attempts occurred in 10 (50%) student attempts, five (23%) resident attempts, and nine (45%) staff attempts with both restricted jaw opening and neck extension and in one (5%) student and two (9%) resident attempts with restricted jaw opening. There was no difference in the rate of failed intubations across experience levels (p=0.33). All intubation attempts with normal settings and only restricted neck extension settings were successful.

Incisor dislodgement

Dislodgement of the mannequin’s removable upper incisors never occurred in the student or attending physician groups. However, dislodgement occurred in the resident group in one direct laryngoscopy (5%) at normal mobility setting, one (5%) with restricted jaw opening, and three (14%) with restricted neck and jaw mobility (p=0.008).

## Discussion

While multiple studies to date have examined forces applied to the oral cavity and oropharynx during direct laryngoscopy, these data represent the first description of forces compared across learners of varying experience during direct laryngoscopy. As anticipated, laryngoscopists of all experience levels demonstrated increased force application to the incisors and tongue as neck and jaw stiffness increased and overall higher forces on the incisors compared to the tongue base at all difficulty levels. More experienced practitioners applied proportionally less force on the incisors compared to less experienced groups. Notably, experienced otolaryngologists demonstrated preferential force application to the distal end of the laryngoscope centered at the tongue base. Interestingly, intermediate learners (i.e., residents) did not hold to this trend, exhibiting increased force application at the tongue base compared to novices (i.e., medical students). The increased force on the tongue base may represent a characteristic profile in direct laryngoscopy skill acquisition as practitioners learn to avoid excessive pressure on dentition but have not yet learned to leverage significant pressure on the tongue base to achieve an optimized view of the vocal folds. Furthermore, these data suggest assessing not only raw force values but also relative proportion of forces applied to the incisors and tongue base for an individual may help assess proficiency and progress when learning to perform direct laryngoscopy.

It should be noted that raw forces demonstrated in this study were comparable to those described in multiple previous studies, which reported forces to oropharyngeal soft tissues and incisors in the range of 2-5 lbf and 5-10 lbf, respectively [26,27,29,31]. These studies were performed in both various mannequin simulators and humans during routine operations, lending veracity to these data’s applicability for simulating realistic direct laryngoscopy scenarios.

This study design was limited in that the mannequin design and active measurement of laryngoscopy forces precluded ability to blind participants as to the primary outcome (i.e., force measurement) and variables (i.e., laryngoscopy difficulty). Moreover, this model was limited by force application to the tongue base being approximated by a sensor on the laryngoscope rather than a pressure sensor on the tongue. The decision to utilize a force gauge on the distal portion of the laryngoscope was due to inability to effectively position a force sensor within the tongue without frequent mechanical abrasion to the sensor and accelerated damage to the soft silicone of the simulated tongue, leading to inaccurate readings and frequent sensor failure. Future versions of this model could potentially incorporate an array of force sensors or a linear force sensor along the tongue midline, allowing for a dynamic force measurement of laryngoscopy forces throughout the oral cavity and oropharyngeal soft tissues. However, as currently designed, this model proved extremely robust - during the design and testing, a single mannequin exhibited uvular loss and only minor superficial tears to the silicone tongue, soft palate, and oral commissures after being intubated over 300 times in a period of four weeks. Given the exchangeability of all components of this mannequin, these are not expected to significantly limit the utility or lifespan of this model.

## Conclusions

This study describes a replicable, modifiable model composed of commercially available parts for the measurement of force application to incisors and oropharyngeal soft tissues during direct laryngoscopy. The data demonstrate that the proposed model reliably quantifies forces during intubation. Furthermore, this model identifies patterns in force application across user experience levels, specifically that force on the tongue base increases and force on the incisors decreases with increasing user experience. This model and the insights it provides may be useful for training and proficiency assessment in direct laryngoscopy and difficult airway management.
